# Dielectric coated conductive rod resonantly coupled with a cut transmission line as a tunable microwave bandstop filter and sensor

**DOI:** 10.1016/j.heliyon.2024.e24477

**Published:** 2024-01-11

**Authors:** David Hambaryan, Tigran Abrahamyan, Henrik Parsamyan, Artyom Movsisyan, Bill Minasyan, Hovhannes Haroyan, Arsen Babajanyan, Kiejin Lee, Barry Friedman, Khachatur Nerkararyan

**Affiliations:** a Institute of Physics, Yerevan State University, Yerevan, Armenia; b Department of Physics, Sogang University, Seoul, South Korea; c Department of Physics, Sam Houston State University, Huntsville, USA

**Keywords:** Electromagnetic surface waves, Goubau line, Sommerfeld waves, Bandstop filter, Microwave sensor, Cut transmission line

## Abstract

The resonant interaction of a dielectric-coated conductive rod with the X-band microwave field is investigated. The magnetic field distribution of the Goubau standing radial surface waves was experimentally visualized by using a thermo-elastic optical indicator microscope, and the corresponding electric field distribution was determined via numerical simulations. These field distributions are characterized by a certain pattern of antinodes distinctive for standing waves. An analysis of these field distributions allows one to couple a coated rod with a cut Goubau line. A rod placed in the gap region perpendicular to the Goubau line results in a sharp rejection band in the transmission spectrum which is extremely sensitive to the changes in the surrounding media. The shifting rate of the resonance as a function of the dielectric shell thickness is approximately 1.4 GHz/mm. The *Q*-factor of copper rods depends on their size and dielectric shell thickness. Longer rods with more energy localization areas have higher *Q*-factors, typically 1.7 times higher (12.7 vs. 7.5). Moreover, incorporating a dielectric shell enhances energy confinement and can elevate the *Q*-factor by as much as 22 %. When a 25 mm Cu rod is situated inside a cut Goubau line system, the *Q*-factor values are significantly higher, with a ratio of 275 to 13. With the addition of a dielectric shell, the *Q*-factor can be elevated by 58 %. The versatility of the proposed controllable system makes it possible to tune the operating spectrum towards higher GHz and THz frequencies.

## 1 Introduction

For application of the GHz-THz frequency range, one needs to reduce dielectric and ohmic losses, as well as to weaken the dispersion of the propagating signal. One of the possible ways to solve these problems is using surface waves on wires of a circular cross-section discovered by Sommerfeld. However, the large extension of the Sommerfeld surface waves in the radial direction makes them less practical since to avoid strong field distortion, a large isolated area around the conductor is needed [1]. The range of possible applications of these waves was expanded when Goubau discovered that the lateral confinement of these surface waves can be drastically enhanced by coating wires with a dielectric layer or corrugating the wire surface – configurations that are referred to as a Goubau line [[2], [3], [4]]. Along with the improved field confinement, these communication elements have a number of advantages, such as low losses and dispersion, as well as a very simple technological process of fabrication which make them good candidates for ultra-wideband applications. The need for low-loss and ultra-wideband wave-guiding systems in the GHz and THz regions triggered renewed interest in different types of the Goubau line. The associated problems were extensively revisited within the last decade [[5], [6], [7], [8], [9]] parallel to the rapid development of high-speed broadband communication technologies such as 5G and 6G networks [10]. Note, that in the current data transfer applications, the distances for wave-guiding have been reduced drastically to the dimensions of a single board or integrated. Planar analogs of corrugated Goubau lines combined with conventional microstrip transmission line design were widely applied to low-loss transmission lines in the THz, super- and extremely-high frequency spectra [8,9,11,12]. Another interesting phenomenon associated with these corrugated configurations is that they can support tunable and geometry-controlled surface waves or so-called spoof plasmons high-frequency analogs of well-known optical surface plasmons [13]. Nevertheless, the spoof-plasmon-based transmission lines usually exhibit higher attenuation than Goubau lines [14] and the latter is currently in the further development stage. A number of electronic devices have been proposed based on the concept of the Goubau line, to name a few, wideband transmission lines [4,15] and passive components [16], leaky-wave antennas [14,17,18], frequency-selective configurations [11,12,19], sensors [[20], [21], [22]], etc. In particular, Parker-Jervis et al. designed a tunable bandstop filter in the THz spectrum using two coupled split-ring resonators integrated with a planar Goubau line and achieved a relatively high Q-factor around 60 at about 270 THz [19]. In Ref. [11], the authors engineered narrowband resonances in the transmission spectrum of a planar Goubau line at GHz and THz frequencies by adding metamaterial resonators along the line. The electromagnetic (10.13039/100006138EM) response of all these systems is manipulated by employing individual elements capable of supporting configuration-dependent resonances.

In this paper, we investigate the resonant properties of an individual conductive rod coated by a thin dielectric layer. We examine the distribution of the EM waves in these resonators via thermo-elastic optical indicator microscopy (TEOIM) [23] and computer simulations. In this configuration, the formation of standing waves is due to the unique distribution of radial surface waves which ensure noticeable reflection from the edges of the rod. Easy coupling with the plane EM waves polarized along the rod axis is ensured owing to a relatively weak component of the electric field of surface waves formed along the rod axis.

The article is organized as follows: first, we examine the distribution of the EM waves in the above-mentioned resonators via TEOIM and computer simulations. This approach reveals the standing-wave origin of the modes and establishes efficient mechanisms of the excitation of such resonators. In the second part, we study the integration of the dielectric-coated cylindrical conductive resonator with the Goubau line. The presence of the dielectric-coated conductive rod yields a narrowband resonance that can find practical application as a sensitive system for changes in ambient conditions.

## 2 Materials and methods

**Fig. 1:**
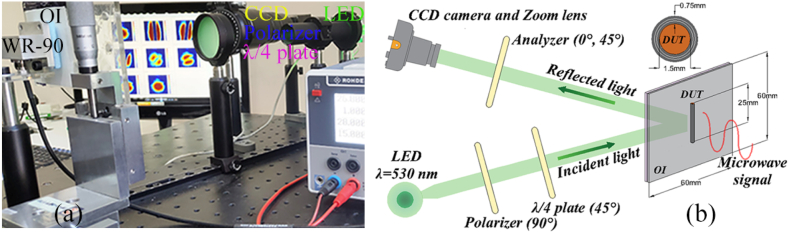
(a) Photo and (b) The schematic of TEOIM experimental setup with OI and DUT, the cross-section of DUT: Cu rod with surrounding dielectric media.

The visualization of microwave magnetic near-field (MMNF) by the TEOIM revealed unique properties of the interaction of a finite-length conductive rod with microwaves [23,24]. Particularly, Cu rods with a height of multiple half-wavelengths support Sommerfeld standing waves formed on the Cu/air interface. The photo of the experimental configuration for TEOIM with rod is shown in Fig. 1 (a). Although such surface waves are easily excited by a plane wave polarized along the rod axis, radiation losses are large. A conductive rod coated by a dielectric layer will provide improved field confinement and smaller radiation losses enabling the use of such elements as radial-wave resonators with controllable parameters. The Cu rod's coating consists of a heat-shrink tube composed of a polymer that contracts when exposed to heat. This tube envelops the Cu rod when heated, conforming to its shape and forming a uniform dielectric cover. Note that our measurements (not shown here) indicate that this polymer material exhibits minimal losses in the X-band ($\varepsilon^{\prime \prime }=0.05\ll \varepsilon^{\prime }=4)$. In other words, it does not contribute to a reduction in the *Q*-factor of the entire system due to self-losses.

**Fig. 2:**
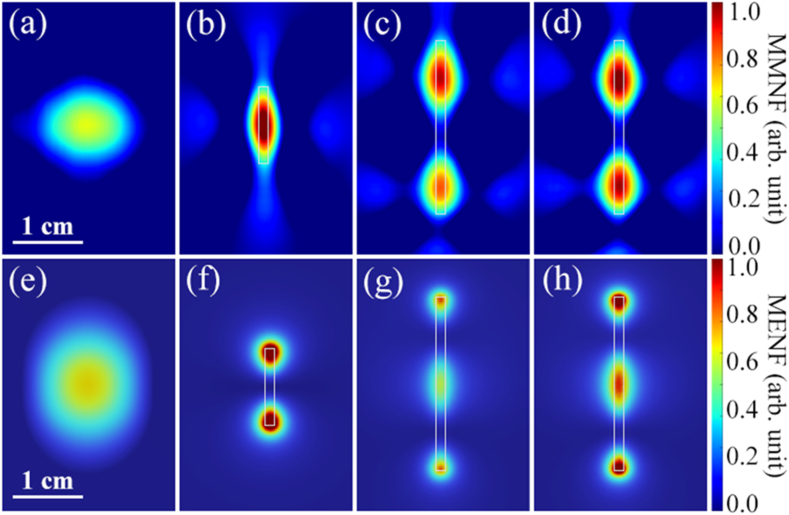
Distributions of the intensity of electromagnetic field components (free field: FF) near the samples at 10 GHz. Тhe distribution of the magnetic field in-plane component of the (a) incident wave, (b–c) bare Cu rods with a height of 11 mm and 25 mm, and (d) 25-mm-height Cu rod covered by an insulator layer of a thickness of 0.5 mm, all obtained via TEOIM. (e–h) The corresponding simulated electric field distributions within a plane 1 mm away from the DUT. All rods have a diameter of 1.5 mm. The grey rectangles represent the contours of Cu rods.

The X-band (8–12 GHz) microwave response in the near-field region of the device under test (DUT) was determined via TEOIM. The schematic of the experimental setup and configuration of the OI with DUT is presented in Fig. 1 (b). This recently suggested technique of the visualization of the electric and magnetic fields is based on the coupling of the optical and microwave fields by making use of an optical indicator (OI) [23]. The OI consists of a borosilicate glass coated by a 150 nm-thin ITO layer. The indicator is illuminated by a circularly polarized optical beam and the structural d in the optical indicator arising due to the microwaves causes changes in the polarization upon the reflection from the indicator. Analyzing these changes allows us to eventually retrieve the magnetic field distributions. It is worth noting that the TEOIM system allows visualization only of the in-plane component of the magnetic field. An open-ended WR-90 waveguide was used as a microwave source whose Gaussian-like magnetic near-field distribution at the indicator surface is shown in Fig. 2 (a). The distance between a sample and a waveguide is 5 mm. Each DUT is oriented such that the axis is parallel to the incident microwave electric field polarization.

## 3 Results and discussion

First, we experimentally investigated the distribution of in-plane components of the MMNF of a dielectric-coated Cu rod under 10 GHz microwave irradiation. The results are illustrated in Fig. 2 (b) and (c) for rods with the same diameter of 1.5 mm and height of *h* = 11 mm and *h* = 25 mm. It is seen that the MMNF distributions along both rods have certain similarities. Particularly, 1) field inhomogeneity having the form of antinodes appears in the vicinity of the rods, 2) for a rod with the given height such distribution of the magnetic field occurs only at certain resonant frequencies, and 3) the wave field concentrated around the rod is noticeably increased.

Fig. 2 (d) depicts the distribution of the in-plane component of the MMNF of a Cu rod covered by a thin homogeneous layer of polymer with a microwave dielectric permittivity of about 4. The core rod is identical to the case presented in Fig. 2(c) and the thickness of the polyethylene layer is 0.5 mm.

For the complete investigation of the resonant excitation of the standing wave along the rod and its characteristics, it is also important to investigate the electric field distribution. To validate the experimental results of the visualized fields, we carried out a COMSOL Multiphysics three-dimensional (3D) full-wave numerical analysis based on the finite element method. All the geometrical parameters and incident wave characteristics such as input power and exciting mode completely correspond to the experiments. Particularly, the simulation model includes an open-end WG-90 waveguide with propagating fundamental TE mode. The distance between the open end of the waveguide and the sample surface is 5 mm. The entire model was surrounded by a sphere with the outermost region set as a perfectly matched layer to eliminate the back-scattered signals. The simulated electric field distributions of the waveguide field and coated rod are shown in Fig. 2 (e) and (f)–(h), respectively. The simulated microwave electric near-field (MENF) distributions corresponding to the DUT in the first row are shown in Fig. 2(e–h). A characteristic spatial shift is seen between the antinodes of the electric and magnetic components of the surface standing wave. The presence of the electric field concentrations around the rod edges is also notable.

**Fig. 3:**
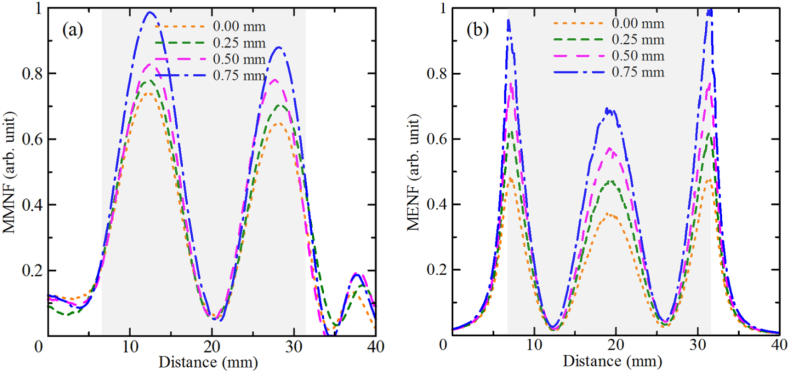
Cross-sectional profiles of the (a) measured MMNF intensity and (b) simulated MENF intensity along the rod axis for a dielectric-coated Cu rod (1.5 mm diameter and 25 mm length) with various thicknesses of the dielectric shell media: 0–0.75 mm. The highlighted grey area corresponds to the Cu rod.

In general, the resonant Sommerfeld standing waves formed on the surface of a bare Cu rod undergo rather large radiation losses. However, the presence of an insulator layer improves the impedance matching between the incident waves in the air and the rod and, thus, increases the coupling efficiency, resulting in an increase in the wave field. One sees that increasing the thickness of the insulating shell from 0 (bare Cu) to 0.75 mm noticeably increases the wave energy localized near the resonator, namely by about 33 % for a resonator with a dielectric layer of *t*_*d*_ = 0.75 mm in contrast to a bare Cu rod. Since the standing waves are formed on the surface of a rod, fixing the rod height and coating it with a dielectric shell led to a shift of the resonance value satisfying the condition of the standing wave formation. This becomes more evident when one looks at the cross-sectional profiles of the MMNF (Fig. 3 (a)) and MENF (Fig. 3 (b)) along the surface line parallel to the rod axis for the experimentally measured magnetic field and numerically simulated electric field intensities, respectively.

**Fig. 4:**
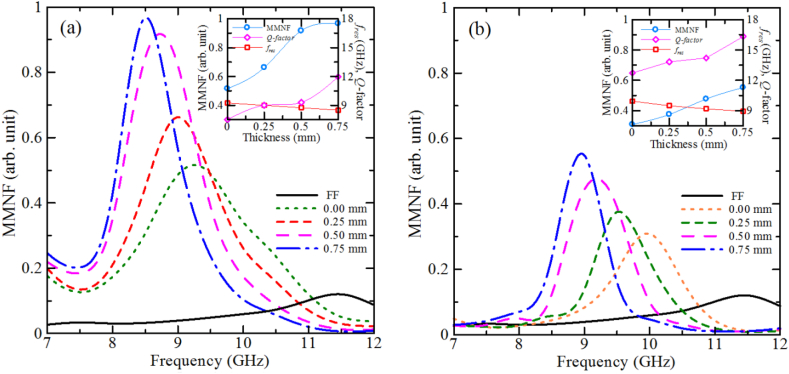
Frequency responses of MMNF of Cu rods with a diameter of 1.5 mm and a length of (a) 11 mm and (b) 25 mm. The thickness of the dielectric layer is varied from 0 up to 0.75 mm. The black line stands for the free field (FF) frequency response of the system without a sample. Insets show the corresponding dependence of the magnitude, resonant frequency, and *Q*-factor of MMNF on the thickness of the dielectric shell.

The resonant frequency of finite-length cylindrical conductive resonator decreases due to coating the rod with a dielectric shell as shown in Fig. 4 (a) for 11 mm and Fig. 4 (b) for 25 mm long rods. Thickening of the dielectric shell is also followed by a narrowing of the resonant line width and, thus, by an increase of the *Q*-factor of the resonator.

The experimentally derived shift of the resonant frequency is an almost linear function of the thickness of the dielectric layer for both investigated rods with a height of 11 mm and 25 mm, as seen in the insets in Fig. 4 (a) and Fig. 4 (b) (red lines). A larger frequency shift is observed for a 25 mm rod where the resonant frequency is decreased from 10 GHz for a bare Cu rod to 9 GHz for a rod with a 0.75 mm-thick insulator layer (1 GHz). Under the same conditions, the resonant frequency shift for an 11 mm long rod is about 0.75 GHz. On the other hand, the dependences of the MMNF magnitude as a function of the dielectric layer thickness are different. In particular, the MMNF magnitude gradually increases and saturates at thicker dielectric shells for an 11 mm rod, whereas for the 25 mm rod, it is an approximately linear function. Such a difference can be attributed to the specifications of the surface modes of each rod. The surface mode of a shorter rod is dipole-like, as seen in Fig. 2 (f), meaning that the wave field has a radiating nature. On the other hand, from the distribution of the electric field component of the surface waves for a 25 mm long rod it follows that the field has a quadrupole-like nature as shown in Fig. 2 (g) and (h). For this case, the total moment is a superposition of two dipole moments in opposite directions formed at the lower and upper half of the rod resulting in much less radiation than its dipole counterpart.

It is noteworthy that the approaches of resonant excitation of standing Sommerfeld waves in 11 and 25 mm long rods by an incident Gaussian beam are different. The incident microwave field polarized along the rod axis induces currents hence ensuring coupling with the longitudinal component of the Sommerfeld wave. Thus, the mode excited in an 11 mm long rod has a half-wavelength, whereas the wavelength of the mode in the 25 mm long rod is around the incident wavelength. When the center of the incident Gaussian beam matches with the rod center, the only 11 mm long rod is effectively excited, while the other rod practically does not respond. However, the latter is effectively excited only when there is an l/4 shift between the rod center and the incident Gaussian beam. In the case of the 11 mm long rod, the longitudinal component of the electric field and the magnetic field (see Fig. 2) are localized at the rod center and ensure efficient interaction with the incident field. On the other hand, the radial component of the standing wave electric field, which noticeably exceeds the axial component, along with the surface charges, is concentrated at rod edges with opposite directions. Fig. 2 (f) illustrates the square of the magnitude of this component. On the contrary, the distribution centers of the longitudinal component of the electric field and magnetic field of the 25 mm long rod are shifted from the rod center by a quarter (Fig. 2(c)) and have opposite directions along with the surface currents. Meanwhile, the radial component of the standing wave electric field and surface charges are localized at the rod center with one sign and at the edges with another.

A rather strong improvement of the confinement of the Sommerfeld radial surface waves of a Cu rod due to the dielectric shell is also confirmed by the evaluation of the MMNF intensity as a function of the frequency for resonant elements with different shell thicknesses. The thickness of the dielectric layer was increased from 0 (bare rod) to 0.75 mm. The experimentally obtained results are plotted in Fig. 4. It is seen that the MMNF intensity of a rod with 0.75-mm-coating approximately doubles compared to that of a bare Cu rod.

**Fig. 5:**
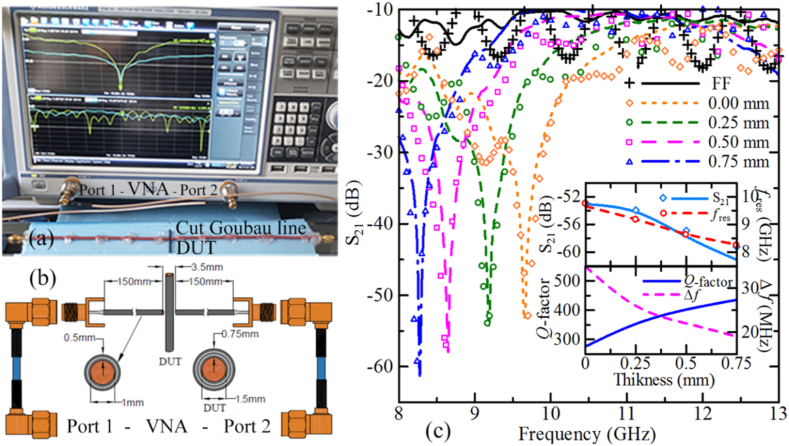
(a) Photo and (b) schematics of VNA testing setup and the DUT integrated with a cut Goubau line. (c) Microwave transmission *S*_21_ coefficient profiles for Cu rod (1.5 mm diameter and 25 mm length) with different thicknesses of surrounding dielectric media: 0–0.75 mm. The black line stands for the system without a Cu rode, i.e., cut Goubau line only (FF). The upper inset shows the dependence of *S*_21_ minima and resonant frequency on the thickness of the dielectric shell. Simulation data is depicted through symbols, while experimental data is represented by lines. The lower inset shows the dependence of system *Q*-factor and bandwidth on the thickness of the dielectric shell.

Now we turn to possible practical applications of these effects. Integration of this resonant element with a transmission line can open up the possibilities of using the resonant properties of surface waves. To do this, we chose a Goubau line representing a transmission line based on a conductive cylindrical core and a dielectric coating. Cu wires having a core radius of 0.5 mm and covered by a polyethylene layer of a thickness of 0.25 mm were used as Goubau lines in experiments. A small gap is created in the Goubau line and the resonant element is placed within the gap perpendicular to the line such that the minimum distance between the edges of both sections of the Goubau line and the center of the resonant element between them is kept at 1.75 mm. The input and output ports of the Goubau lines were connected to the Rohde & Schwartz ZNB20 vector network analyzer (VNA). The measurement configuration and schematic sketch of the experimental setup are shown in Fig. 5 (a) and (b). The transmission coefficient *S*_21_ of such a configuration consisting of the cut Goubau line coupled with the 25-mm-long cylindrical core-shell Cu-polymer resonant element, when the thickness of a dielectric shell was varied from 0 (bare Cu rod) to 0.75 mm, is shown in Fig. 5 (c). Here, the experimental and simulation data are compared.

The Goubau line can be resonantly coupled with a cylindrical core-shell element through the electric component of the wave field. This is due to the fact that an evaluation electric field will be concentrated at the edge of the Goubau line, as seen in Fig. 2 (f)–(h). Electric field distribution of surface waves for a 25 mm length rod with and without a dielectric cover shows an antinode of the standing waves at the center of the rod. This line of observation allows one to establish the efficient configuration of the coupling of the dielectric-coated conductive rod with a cut Goubau line. As a result, a sharp dip in the transmission spectrum is observed in such a system.

Similar to the previous experiments here again increasing the dielectric shell thickness results in a shift of the resonant peak position towards lower frequencies, and, at the same time decrease in the full width at half maximum (FWHM) of the resonance curve as shown in Fig. 5 (c). However, in the second experiment, the resonance FWHM is noticeably smaller being of the order of 0.3 GHz for a rod with a 0.75 mm dielectric shell and 0.4 GHz for a bare Cu rod. The shifting rate of the resonant frequency as a function of the dielectric shell thickness is estimated to be 1.4 GHz/mm, as shown in the upper inset of Fig. 5 (c). The sharpest decrease in the transmission coefficient is about 10 dB/mm. Thus, varying the thickness of the dielectric shell by 1 μm will lead to a change of around 0.01 dB and 1.4 MHz for *S*_21_ and resonant frequency, respectively, based on the signal-to-noise ratio (SNR) of the VNA used in the measurements.

**Fig. 6:**
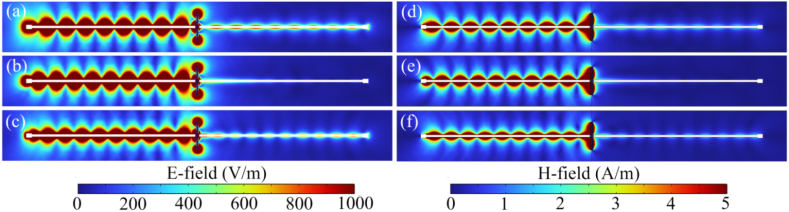
(a–c) Electric and (d–f) magnetic field distributions for bare Cu rod with a height of 25 mm and covered by an dielectric layer of a thickness of 0.5 mm at (a,d) 8.1 GHz, (b,e) 8.6 GHz, and (c,f) 9.1 GHz.

In contrast to the *S*_21_, the *S*_11_ of the system remains relatively unchanged with variations in surrounding dielectric thickness (date not provided here). The maximum shift in *S*_11_ resonant frequency is 0.1 GHz, while for *S*_21_, this shift is 1.5 GHz. The optimization of this resonant system is geared towards controlling wave field transmission rather than reflection. This assertion is correlated by simulation data depicting E-field and H-field distributions shown in Fig. 6. The electromagnetic field distributions are illustrated for a shell thickness of 0.5 at resonant (8.6 GHz) and at left-shift (−0.5 GHz) and right-shift (+0.5 GHz) frequencies. Both electric (Fig. 6(a–c)) and magnetic (Fig. 6(d–f)) field distributions indicate that the wave field intensity passing through the Cu rod covered with dielectric layer is minimal at 8.6 GHz. The resonant behavior of the simulated electric and magnetic field distributions, along with the numerically calculated dependence of the *S*_21_ coefficient on the frequency at the cut Gaboue line with the presence of a Cu rod near the gap, is evident. The pronounced resonance in the *S*_21_ parameter is explained by the antiphase response of the Cu rod near resonance conditions, as illustrated in Fig. 4. When this response opposes the phase of the propagated surface field in the left Goubau line (to which the source is connected), it satisfies the standing wave condition. As a result, there are no conditions for energy transfer to the right line, leading to a significant decrease in the *S*_21_ at resonant frequency.

Overall, coupling the rod-resonator (either with or without a dielectric layer) with a cut Goubau line results in certain characteristics. First, the *Q*-factor (defined by *Q* = *f*_*res*_/Δ *f*) of such a coupled system essentially increases in contrast to an individual rod-resonator excited by a waveguide. In particular, evaluation of the *Q*-factor using the spectral response of *S*_21_-parameters of the 25 mm long rod-resonator coupled with a Goubau line, shown in Fig. 5 (c), indicates that the *Q*-factor increases from 275 for the system with a bare rod up to 435 for that with 0.75 mm thick dielectric coating. The influence of the dielectric shell thickness on *Q*-factor, *S*_21_ minimum, resonance frequency, and bandwidth are summarized in Fig. 5 (c) inset top and bottom plots. It is evident that as the length of the copper rod decreases, the resonant frequency of the system shifts towards the higher frequency side by approximately 2 GHz for a 25 mm-length Cu rod, with a corresponding reduction in the magnitude of *S*_21_ by around 6 dB and *Q*-factor by 2 times (the data is not shown here). As the length of the Cu rod is further reduced, the *S*_21_ signal becomes weaker and the resonant behavior disappears. Based on our findings, we propose that the absence of Sommerfeld radial surface waves in the short conductive rod is attributed to its low effective coupling with the Goubau line. Secondly, the system exhibits narrowband frequency selectivity (<20 MHz). Thus, it can serve as a controllable bandstop filter, enabling manipulation of bandwidth, slope, and *Q*-factor, as well as a sensitive structure for real-time monitoring of environmental parameters, such as electromagnetic and material characteristics and temperature. The resonant coupling of a finite-length conductive rod with a cut Goubau line yields a high degree of selectivity concerning surrounding media and conditions within the GHz to THz frequency range. Compared to the previous setups [11,12,and19]], which proposed a planar Goubau line integrated with split-ring resonators in a planar metastructure with a specific geometry to absorb energy at a precise frequency, leading to narrow-band resonances in the transmission spectrum, the current study explores a simpler structure with improved system performance with improved *Q*-factor of 273. Those studies examined two split-ring configurations for GHz and THz frequencies, achieving *Q*-factors of approximately 11 at 16 GHz, 10 at 0.315 THz [11], and almost 19 at 0.272 THz [19]. Noticeably, the resonance of the surface standing waves can be tuned towards higher GHz and THz frequencies through a careful choice of the geometrical and material parameters of the resonator and the dielectric shell. Analogous behavior can be expected when altering the complex dielectric permittivity of the shell material by external stimuli, which can establish a basis for using such a system as a controllable filter, as well as exploiting the sharp-resonance-driven characteristics for sensing applications.

## 4 Conclusions

A thin conducting rod covered by a dielectric layer can serve as a resonant element supporting standing surface Sommerfeld waves in the microwave X-band. Coupling such a resonator with a cut Goubau transmission line allows one to engineer a transmission spectrum characterized by sharp dips at certain resonant frequencies. The position of the dip can be effectively tuned by varying the thickness of the dielectric shell. In particular, the shifting rate of the resonant frequency as a function of the dielectric shell thickness is estimated to be 1.4 GHz/mm and the sharpest decrease in the transmission coefficient is about 10 dB/mm. Utilizing a finite-length conductive rod resonantly coupled with a cut Goubau line structure results in a significant increase in *Q*-factor (by 22-fold) and narrowband (<20 MHz) frequency selectivity. Furthermore, such a simple configuration allows easy rescaling of the geometrical dimensions and shifting the resonances to higher frequencies. Due to the sharp resonances, the electromagnetic response of the system will be very sensitive to external stimuli, enabling applications of such a system as a controllable bandstop filter or sensor.

## Data availability statement

Data will be made available on request.

## CRediT authorship contribution statement

**David Hambaryan:** Validation, Investigation. **Tigran Abrahamyan:** Validation, Methodology, Investigation. **Henrik Parsamyan:** Writing – original draft, Software, Methodology. **Artyom Movsisyan:** Software, Investigation. **Bill Minasyan:** Investigation. **Hovhannes Haroyan:** Writing – review & editing, Software, Methodology. **Arsen Babajanyan:** Writing – review & editing, Visualization, Methodology, Formal analysis. **Kiejin Lee:** Writing – review & editing, Resources, Methodology. **Barry Friedman:** Writing – review & editing. **Khachatur Nerkararyan:** Writing – review & editing, Writing – original draft, Validation, Supervision, Resources, Formal analysis, Conceptualization.

## Declaration of competing interest

The authors declare that they have no known competing financial interests or personal relationships that could have appeared to influence the work reported in this paper.
